# Evaluating Bidirectional Predictive Pathways between Dietary Restraint and Food Addiction in Adolescents

**DOI:** 10.3390/nu15132977

**Published:** 2023-06-30

**Authors:** Julia M. Rios, Martha K. Berg, Ashley N. Gearhardt

**Affiliations:** Department of Psychology, University of Michigan, 530 Church St., Ann Arbor, MI 48109, USA; bergmk@umich.edu (M.K.B.); agearhar@umich.edu (A.N.G.)

**Keywords:** food addiction, dietary restraint, adolescence, longitudinal analysis

## Abstract

The relationship between food addiction, an important emerging construct of excessive eating pathology, and dietary restraint has yet to be fully understood. Eating disorder models commonly posit that dietary restraint exacerbates loss of control eating (e.g., binge episodes) and may also play a causal role in the development of food addiction. However, dietary restraint as a reaction to consequences of food addiction (e.g., uncontrollable eating or weight gain) represents another plausible pathway. Existing studies indicate that the association between food addiction and dietary restraint may be more significant during adolescence than adulthood, but are limited by cross-sectional study designs. A longitudinal study using an adolescent sample is ideal for investigating potential pathways underlying links between food addiction and dietary restraint. This study examined temporal pathways between food addiction and dietary restraint in a sample of one hundred twenty-seven adolescents (M = 14.8, SD = 1.1) at three timepoints spanning two years. This is the first study to examine longitudinal cross-lagged panel associations between food addiction and dietary restraint. In this adolescent sample, food addiction significantly predicted future dietary restraint (b = 0.25, SE = 0.06, *p* < 0.001), but dietary restraint did not significantly predict future food addiction (b = 0.06, SE = 0.05, *p* > 0.05). These findings support the theory that dietary restraint may be a reaction to deleterious effects of food addiction during adolescence.

## 1. Introduction

Food addiction is an emerging classification of compulsive overeating, characterized by “excessive overeating of high-calorie food accompanied by loss of control and intense food cravings” [1,2]. Alongside empirical support for the concept of food addiction, remaining questions regarding its mechanisms and clinical utility have stimulated scholarly debate. A leading critique of the food addiction construct is that existing models do not adequately account for contributions of dietary restraint [3]. Dietary restraint has often been defined as a self-imposed restriction of food intake in order to lose weight or avoid weight gain [4]. However, empirical evidence suggests that individuals who report high levels of dietary restraint do not always appear to be actually reducing or restricting caloric intake [5,6]. More recently, dietary restraint has been better understood to also encompass cognitive efforts to reduce overall intake or avoid certain types of food regardless of success [7,8].

Eating disorder models have traditionally held that maladaptive (e.g., rigid or excessive) dietary restraint is a critical antecedent to binge eating [9,10]. According to many eating disorder models, dietary restraint creates a state of physiological and psychological deprivation which is difficult to maintain and ultimately induces pathological overeating [11]. In contrast with traditional eating disorder models, food addiction models do not currently centralize dietary restraint as a causal etiological factor [9,12,13]. Because food addiction and binge eating share key features (e.g., loss of control eating and food cravings [14]), it stands to reason that findings on dietary restraint and related eating disorders (e.g., BED and BN) provide valuable theoretical insight regarding how dietary restraint may influence similar eating pathology in food addiction. Based on the dietary restraint literature in eating disorders, it is plausible that dietary restraint may also contribute to food addiction pathology or occur as a reaction to negative consequences of food addiction. However, addictive eating and binge eating are nonidentical pathologies and restraint is not a central causal feature in known addiction pathways (e.g., substance use disorders [12]). Numerous prior cross-sectional studies have failed to find evidence for an association between food addiction and dietary restraint in adults [15,16,17], though findings have been mixed in a small number of international studies [18,19]. Thus, it is also possible that dietary restraint may not be strongly associated with food addiction, highlighting the need to examine their distinct relationship. However, the existing literature on associations between food addiction and dietary restraint is overall sparse, and potential relations or directional pathways have received limited empirical investigation. Therefore, this study aims to shed light on potential temporal pathways and directionality in the relationship between these constructs.

Adolescence is a potentially key period during which to investigate the relationship between food addiction and dietary restraint. Adolescence is a high-risk period for both the emergence of dieting behaviors and heightened vulnerability to addictive behaviors [20,21]. However, few existing studies, to our knowledge, have explored food addiction and dietary restraint in adolescents. In one study, food addiction symptoms assessed by the dimensional Yale Food Addiction Scale for Children 2.0 (dYFAS-C 2.0), a version of the YFAS adapted to reflect age-appropriate symptoms (e.g., problems at school instead of work) and reading level [22], were positively correlated with dietary restraint scores on the DEBQ-R (*r* = 0.32 [23]). A second study showed that YFAS scores were significantly correlated with the Three Factors Eating Questionnaire dietary restraint subscale in Turkish adolescents (OR = 1.01 [24]). Of note, the effect size of these associations were small. Nonetheless, these studies suggest that food addiction and dietary restraint may be related in adolescents but are limited by cross-sectional research design.

According to restraint-based theories for eating pathology, these prior findings could be interpreted as evidence that dietary restraint increases the risk for addictive eating behaviors. However, these results could also signify that adolescents with higher propensity for addictive eating may be more likely to engage in dietary restraint as a reaction to patterns of overconsumption and possible weight gain. If dietary restraint is a stronger predictor of future food addiction, this may support restraint-based theories for a causal role of dietary restraint. Alternatively, if food addiction is a stronger predictor of future dietary restraint, this may support a model of food addiction in which risk is primarily underlied by exposure to HP foods, and dietary restraint occurs as a consequence, rather than a cause, of addictive eating. If neither dietary restraint nor food addiction predicts longitudinal change in either construct, this may indicate that dietary restraint is a less relevant construct in food addiction than binge-type eating disorders.

The limitations of cross-sectional prior studies make it difficult to speculate on the nature of the relationship between food addiction and dietary restraint over time. This study aims to determine whether food and dietary restraint are correlated in a sample of adolescents (*N* = one hundred twenty-seven) in a longitudinal design over a two-year period of repeated assessment. This will increase understanding of the temporal associations and possible directionality between food addiction and dietary restraint that appears during adolescence.

## 2. Materials and Methods

### 2.1. Participants

**Recruitment**. As part of a larger longitudinal study examining adolescent eating behavior and responsivity to food advertisements (Project Media), adolescent participants (i.e., 13–16 years of age) were recruited from southeast Michigan using print and online advertisements. A parent or guardian provided written informed consent and adolescents provided written informed assent prior to enrollment. Adolescents (*N* = 127), ranging from 13 to 16 years of age (M = 14.8, SD = 1.1), were recruited for the full study. The dimensional YFAS-C 2.0 (dYFAS-C 2.0) was added to the questionnaire battery later in data collection, and 127 participants completed the measure at the initial wave of data collection (Time 1). Participants completed self-report measures at baseline (Time 1) and follow-ups after one year (Time 2) and two years (Time 3). Participants provided demographics at baseline (Time 1; participant descriptives are summarized in Table 1). Due to the sensitive nature of some of the self-reported content, additional protocols were implemented to reduce potential bias and increase validity of responses. Participants were provided a private space to complete all measures and were informed that all researchers were blind to their de-identified responses. This study was approved by the University of Michigan Institutional Review Board (IRB) and complied with the ethical standards of the APA (APA, 2013).

**Inclusion and Exclusion Criteria**. Due to the aims of the larger study investigating eating behavior and reward response to food marketing in adolescents, participant exclusion criteria included the following factors known to influence reward functioning: (1) a history of or a current eating disorder diagnosis, (2) current mood, anxiety, trauma, or psychotic disorders, (3) current prescription for a psychotropic medication, and (4) underweight BMI status.

### 2.2. Measures

**Demographics and Anthropometry**. Participants were asked to complete a demographics questionnaire at the first study visit (Time 1). Participants were asked to self-report their date of birth (which was used to calculate age in months), race, gender (as male, female, other gender identity, or prefer not to identify), and parental education level. Participant height and weight measurements were taken at the first study visit (Time 1). Participants were asked to remove any shoes, hats, and outerwear. Participant heights and weights were taken twice to confirm accuracy. Participants were weighed to the nearest 0.1 kg using a Detecto Portable Scale. If weights differed by more than 0.1 kg, the measurements were repeated. Participant height was measured using an O’Leary Acrylic Stadiometer to the nearest 1 cm. If height measurements differed by more than 1 cm, the measurements were repeated. Participant BMI z-scores were calculated using percentiles determined by the Center for Disease for Control’s assessment for children and teens [25]. Participant demographics and anthropometry measures are summarized in Table 1.

**dYFAS-C 2.0**. The dYFAS-C 2.0 is a 16-item self-report measure that operationalizes food addiction characteristics in children and adolescents based on the same DSM-5 criteria for substance use disorders as the YFAS ([16,23]. When completing the dYFAS-C 2.0, participants are instructed to think about foods high in refined carbohydrates and/or fats, as these foods have been most evidenced in food addiction [26]. All items are reported on a 5-point Likert scale (from 0 = never to 4 = always). Prior research suggests that problematic substance use in adolescence is more accurately conceptualized as a continuous rather than a categorical syndrome [27]. Thus, the dYFAS-C 2.0 utilizes a dimensional scoring approach. Item scores are summed with higher scores indicating more severe food addiction. The dYFAS-C 2.0 demonstrates good convergent and incremental validity, as well as internal consistency [23]. In the current sample, dYFAS-C 2.0 scores demonstrated good internal consistency (α = 0.90).

**Dutch Eating Behaviors Questionnaire Restraint Subscale**. The Dutch Eating Behaviors Questionnaire Restraint Subscale (DEBQ) is a 33-item self-report survey designed to capture various aspects of eating style including external eating, emotional eating, and restrained eating. The 10-item restrained eating subscale (DEBQ-R) measures intentions and attempts to reduce food intake or to avoid certain food types. All items were reported on a 5-point Likert scale (from 1 = never to 5 = very often). Scores on the DEBQ-R reflect the average of all items, with higher scores indicating a greater degree of dietary restraint. The DEBQ demonstrates good predictive validity and internal consistency in adults [28,29], as well as adolescents [30,31]. In the current sample, DEBQ-R scores demonstrated excellent internal consistency (α = 0.93).

### 2.3. Data Analytic Plan

Statistical tests were completed using R version 4.1.2 and the *lavaan* package [32]. Preliminary analyses were completed to verify that these data did not violate assumptions for cross-lagged panel analysis including normality, stationarity, and synchronicity [31]. Individuals who completed all measures at all time points (n = 91) did not significantly differ from individuals who did not complete measures at Time 2 and/or Time 3 of the study (n = 36) on any of our variables of interest (i.e., food addiction or dietary restraint, all *p* > 0.05), covariates (i.e., age, gender, or BMI z-score, all *p* > 0.05), or demographics (i.e., race or parental education; all *p* > 0.05). Full information maximum likelihood estimation was also used to maximize sample size, given an assumption that all missing data was missing at random [33]. Prior to analysis, outlier values from both primary variables (food addiction and dietary restraint) were winsorized [34], and both variables were standardized.

**Figure 1:**
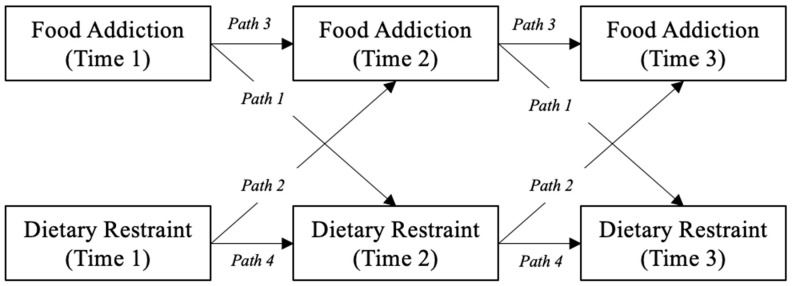
Path Diagram for Unadjusted Cross-lagged Panel Analysis between Food Addiction and Dietary Restraint.

Temporal associations between food addiction and dietary restraint were examined using a cross-lagged panel design across three waves (Time 1, Time 2, and Time 3). To control for possible confounding effects of differences in baseline age, gender, or BMI z-score [35,36], these were considered as covariates in the model. Age and BMI z-score variables were mean-centered, and gender variable was contrast-coded. Zero-order correlations among variables of interest and covariates were tested at Time 1, Time 2, and Time 3. Inclusion of covariates did not change the patterns of significance for any findings in the cross-lagged panel analysis but did result in poorer model fit (unadjusted CFI = 0.95, SRMR = 0.054, adjusted CFI = 0.86, SRMR = 0.14). Thus, for ease of interpretation and improved model fit, results and figures reported here reflect the unadjusted structural equation models (SEM; see Figure 1). The model simultaneously estimated the cross-lagged relationships between the two variables, as well as auto-regressive paths for each variable across time. All effects were assumed to be constant across the three time points; therefore, a single estimate was computed for each cross-lagged and auto-regressive relationship, independent of time (see Figure 1 for labeled path diagram). Pathways and results from the adjusted SEM are provided as Supplemental Materials (see Supplemental Materials Figure S1 and Table S2). We also conducted exploratory imputation analyses to account for missingness in the data at Time 2 and Time 3. However, no substantial differences were observed in the correlations or cross-lagged panel analyses between the non-imputed and imputed data sets. Thus, the results from the non-imputed data are reported here.

## 3. Results

Food addiction and dietary restraint were significantly associated with each other at all time points (*r* = 0.34, *p* = <.001; *r* = 0.36, *p* = < 0.001; *r* = 0.47, *p* = < 0.001), and were each associated with age, gender, and BMI z-score at most time points (see Supplemental Table S1).

Cross-lagged panel analysis revealed that food addiction significantly predicted future dietary restraint (b = 0.25, SE = 0.06, *p* < 0.001). Dietary restraint did not significantly predict future food addiction (b = 0.06, SE = 0.05, *p* > 0.05; see Table 2 for all model estimates). In comparing the difference between coefficients for each of the cross-lagged paths, Path 1 (food addiction predicting dietary restraint) was significantly stronger than Path 2 (dietary restraint predicting food addiction; b = 0.18, SE = 0.08, *p* < 0.05). Auto-regressive paths were significant for both food addiction (b = 0.61, SE = 0.05, *p* < 0.001) and dietary restraint (b = 0.59, SE = 0.056, *p* < 0.001) over time, suggesting both constructs showed test-retest reliability over time.

## 4. Discussion

In a longitudinal study of one hundred twenty-seven adolescents, we assessed and compared the strength of predictive pathways between food addiction symptoms and dietary restraint across two years. Cross-lagged panel analyses showed that food addiction significantly predicted future dietary restraint over time. In contrast, dietary restraint did not predict future food addiction. To address our primary research question, we computed the difference between the coefficients for each of the cross-lagged paths. The path for food addiction predicting dietary restraint (Path 1) was stronger than the path for dietary restraint predicting food addiction (Path 2). Auto-regressive paths for food addiction (Path 3) and dietary restraint (Path 4) were both significant, indicating the stability of these predictors over time.

The present findings provide additional support to the existing literature that food addiction and dietary restraint demonstrate some association in adolescents [22,23,24]. While some researchers have thereby speculated that dietary restraint plays a causal role in the development or progression of food addiction [38], these longitudinal findings show that food addiction is a stronger predictor of future dietary restraint than dietary restraint is of future food addiction. This suggests that food addiction may be more likely to emerge prior to dietary restraint, and that dietary restraint occurs as a consequence rather than a cause of food addiction. This finding is consistent with other models of addiction (e.g., substance use disorders) in which individuals exhibit restraint in an effort to control addictive behaviors or substance use [12,13].

This is in contrast to some predominant models for binge eating, which have historically suggested that dietary restraint is a causal preceding factor [9,10]. However, findings from more recent empirical studies on binge eating and dietary restraint have been mixed. While dietary restraint appears to be a relevant factor in binge-type eating pathology for some individuals (e.g., about half of individuals who develop BED [39,40,41]), binge-type eating behaviors are reported to precede dietary restraint for many others [39,42,43]. The present findings suggest that the relationship between food addiction and dietary restraint in adolescence may be more consistent with the subgroup of individuals for whom dietary restraint appears to occur as a reaction to binge-type eating pathology (e.g., in an effort to avoid weight gain [44,45]). It may be that an addictive response to HP foods results in a greater tendency to engage in reactionary dietary restriction (e.g., due to social pressures and beauty ideals about thinness) to offset excessive food intake. A recent study demonstrated that repeated exposure to HP foods in healthy, normal-weight participants led to increased sensitization to the rewarding properties of HP foods and related neurobehavioral dysfunction, such as decreased preference for minimally processed foods and increased consumption of HP foods [46]). Thus, individuals who exhibit addictive eating of HP foods may be more likely to engage in dietary restriction in order to combat increased consumption of HP foods or related weight gain. Future research utilizing latent class growth analyses may provide more specific insights into latent pathways or subgroup differences for associations or temporal relationships among these constructs.

In sum, while these constructs do appear to be related during this stage of development, longitudinal analyses do not support a causal role of dietary restraint in mechanistic models of food addiction. Rather, the present findings provide stronger empirical support for a model of food addiction in which risk may be underlied by alternative factors, such as exposure to HP foods [44,45], clinical co-morbidities and psychological risk factors (e.g., addiction proneness [47]), stronger reward sensitivity [46,48], vulnerability for weight gain [44,45], or addiction risk factors (e.g., family history of addiction [49]). Therefore, evidenced associations between food addiction and dietary restraint in adolescents may reflect attempts to manage an addictive response to HP foods. The present study provides empirical evidence for a direct temporal pathway between food addiction and dietary restraint. It will be important for future research to investigate theoretical risk factors (e.g., proneness to addiction or exposure to HP foods) or mediators (e.g., weight or shape concerns) in the model.

If food addiction is a stronger predictor of future dietary restraint, strategies aimed specifically at ameliorating food addiction symptoms may be most effective for reducing future dietary restraint and any amplifying effects on future food addiction. The implementation of prevention (e.g., health-promoting school and home settings [50]), treatment (e.g., adapted addiction treatment programs, treatment of comorbid psychopathology [51]), and policy (e.g., restrictions on HP food marketing that targets teens [52]) interventions during the crucial stage of adolescence may have substantial benefits for reducing both food addiction and dietary restraint behaviors in adolescents. Currently, the majority of prior research has been dedicated to evaluating the food addiction construct, and much less research has explored or tested intervention approaches. This will be an important future direction for food addiction research.

There is a critical need for research-guided public health recommendations for dietary restraint that address the impairment and distress related to symptoms of food addiction [1] and living in a social environment that stigmatizes weight gain and fatness [53]. Importantly, existing research suggests that not all forms of dietary restraint are associated with equally poor eating outcomes. For example, rigid dietary restraint (e.g., all-or-nothing dieting approach) compared with flexible dietary restraint (e.g., graduated dieting approach) appears to be more strongly associated with binge-type eating when dieting rules are violated [54]. Prior research also shows improvements in eating outcomes (e.g., reduced external eating and emotional eating) when dietary restraint is implemented regularly and proactively (e.g., routine restraint) compared to irregularly and retroactively (e.g., compensatory restraint following diet noncompliance [55]). It is therefore possible that the type of dietary restraint which occurs in reaction to food addiction may have differential impacts on health and well-being. Intervention strategies that promote more flexible and proactive dietary restraining behaviors may have some pro-health utility. However, additional research is needed to better understand which forms of dietary restraint may be most harmful, neutral, or beneficial for promotion of healthy eating behaviors and how this may interact with a propensity for food addiction. It will be critical for future research to explore and develop dietary recommendations for individuals who endorse food addiction symptoms and struggle to control their eating.

This study offers a number of important strengths and contributions to the empirical literature. This is the first longitudinal study design exploring temporal pathways between food addiction and dietary restraint allowing for inferences regarding the nature and directionality of the relationship between these constructs. Furthermore, this study involved adolescent participants and utilized developmentally appropriate psychometrics, which provided an assessment of food addiction and dietary restraint during a key developmental period in which eating pathology and dieting behaviors often emerge [21,56]. However, because this study was limited to a two-year span during adolescence, much remains unknown about early life risk factors and long-term progression of both food addiction and dietary restraint. Existing research indicates that by adulthood, food addiction and dietary restraint have a weaker association [19] or may no longer be associated [16,18,57]. Without longitudinal data spanning adolescence and adulthood, it is difficult to pinpoint why this association seems to diminish over time. Ongoing research is needed to better understand additional factors which may contribute to risk for both food addiction and dietary restraint, as well as how associations between these constructs change throughout various stages of development.

The present study was also limited to a relatively small and well-resourced sample. Additional research is needed to test how these findings may generalize to larger and more diverse populations. The use of a larger sample size would also provide more power to examine possible moderating effects of key covariates (i.e., age, gender, and BMI z-score) or explore latent class analyses to assess for individual differences or subgroups of pathway directionality. This study utilized data collected from a larger study with aims to examine eating behavior and reward response to food marketing in adolescents that excluded participants with psychiatric conditions including eating disorders or underweight BMI status. This allowed us to consider bidirectional effects of food addiction and dietary restraint more precisely, minimizing possible confounding effects of other eating pathology (e.g., binge eating). However, it should be noted this removes participants with the most extreme presentations of dietary restraint and limits the generalizability of the findings.

## 5. Conclusions

The current results utilizing longitudinal data indicate that dietary restraint may be more likely to occur as a reaction or consequence to food addiction symptoms. These findings highlight the potential utility of food addiction as a research target for intervention or prevention efforts towards improving eating behavior. Future research is needed to better understand the bidirectional mechanisms that drive the association between food addiction and dietary restraint in adolescents.

## Figures and Tables

**Table 1:** Adolescent Participant Demographics, Descriptives, and Sample Size at Each Wave (*N* = 127).

	Total (n)	Percent (%)
Gender		
Male	61	48.0
Female	66	52.0
Race		
American Indian/Alaska Native	3	2.4
Black/African American	19	15.0
White	91	71.7
Other	1	0.8
Mixed	8	6.3
Unknown	5	3.9
Parental Education		
Less than High School	15	11.8
High School Degree	5	3.9
Some College	19	15
Associates Degree	11	8.7
Bachelor’s Degree	35	27.6
Advanced Degree	42	33.1
Sample Size at Each Wave		
Time 1	127	100.0
Time 2	92	72.4
Time 3	88	69.3
	Mean (SD)	Range (min, max)
Age (months) at Time 1	177.3 (12.4)	(156.0, 202.5)
BMI z-score	0.95 (0.9)	(−1.2, 2.7)

Note. SD = standard deviation; BMI = body mass index. Data missingness was determined by availability of data for primary variables included in the cross-lagged panel analysis (i.e., food addiction (YFAS) and dietary restraint (DEBQ-R) at each time point).

**Table 2:** Standardized Regression Coefficients for Food Addiction and Dietary Restraint from Unadjusted Structural Equation Models.

							CI (95%)	
Path	Predictor	Outcome	*b*	SE	*z*	*p*	Lower	Upper
1	Food addiction	Dietary restraint	0.25	0.06	4.51	<0.001	0.14	0.37
2	Dietary restraint	Food addiction	0.06	0.05	1.24	0.21	−0.04	0.16
3	Food addiction	Food addiction	0.74	0.05	15.32	<0.001	0.64	0.83
4	Dietary restraint	Dietary restraint	0.58	0.06	10.48	<0.001	0.47	0.68

Model Fit: *X*^2^(8) = 21.14 (*p* < 0.01); SRMR = 0.04; CFI = 0.95. CI = confidence interval. A post-hoc sensitivity analysis using pwrSEM ([37] with ten thousand simulations and a seed of twenty-three indicated that our model had 100% power to detect an effect of this size for Path 1, and 41% power to detect an effect of this size for Path 2. Predictor variables were measured at T1, outcome variables were measured at times T2 and T3.

## Data Availability

The data presented in his study are available on request from Dr. Ashley Gearhardt (agearhar@umich.edu).

## Supplementary Materials

The following supporting information can be downloaded at: https://www.mdpi.com/article/10.3390/nu15132977/s1, Figure S1: Path Diagram for Adjusted Cross-lagged Panel Analysis among Food Addiction, Dietary Restraint, and Covariates (Age, Gender, and BMI z-score); Table S1: Summary of Bivariate Correlations Among Adolescent Food Addiction, Dietary Restraint, and Associated Covariates; Table S2: Standardized Regression Coefficients from Adjusted Structural Equation Models with Covariates.
