# Supplementary material for: Evaluating Bidirectional Predictive Pathways between Dietary Restraint and Food Addiction in Adolescents
Source: Nutrients. 2023 Jun 30;15(13):2977. doi: 10.3390/nu15132977 (PMC10346943; doi:10.3390/nu15132977)
Supplement: Supplementary file 1 [file nutrients-15-02977-s001.zip › nutrients-2422198-supplementary.pdf]

Supplemental Materials

Supplemental Figure S1. Path Diagram for Adjusted Cross-lagged Panel Analysis among Food Addiction, Dietary Restraint, and Covariates (Age, Gender, and BMI z-score)

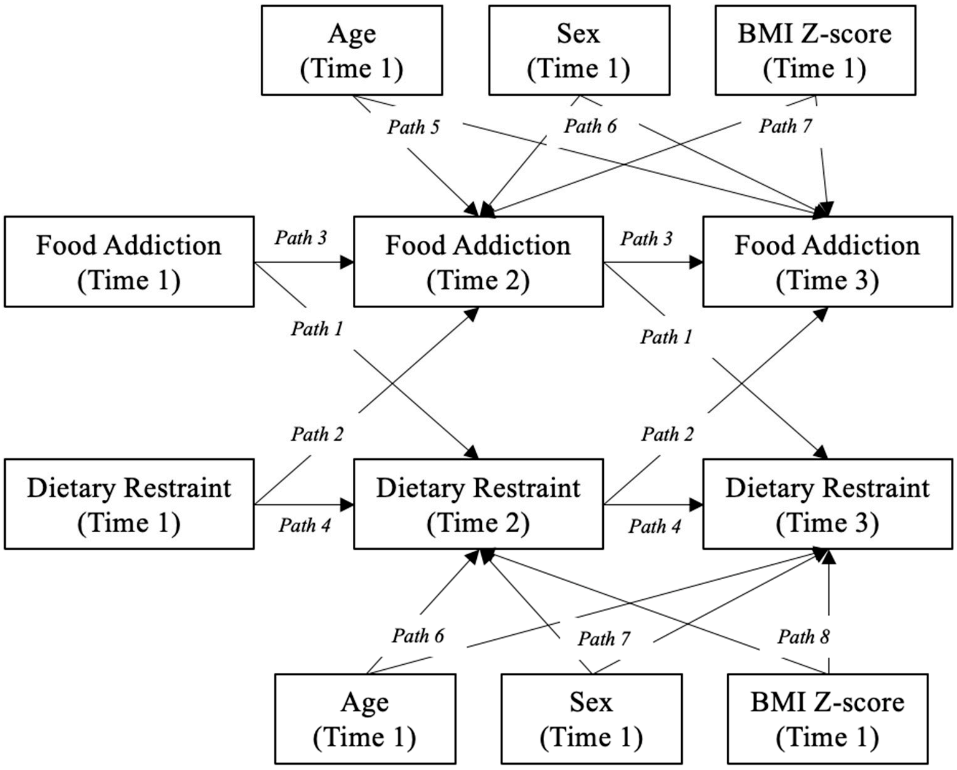

Note. Path 1: food addiction as a predictor for future dietary restraint; Path 2: dietary restraint as a predictor for future food addiction; Path 3: auto-regressive path for food addiction over time; Path 4: auto-regressive path for dietary restraint over time; Paths 5, 6, and 7: confounding effects of covariates (age, gender, and BMI z-Score).

Supplemental Table S1. Summary of Bivariate Correlations Among Adolescent Food Addiction, Dietary Restraint, and Associated Covariates

	Sex	Age	BMI Z-score
Food Addiction (dYFAS-C 2.0)			
Time 1	0.29***	0.27**	0.33***
Time 2	0.30**	0.33**	0.25**
Time 3	0.29**	0.29**	0.24*
Dietary Restraint (DEBQ-R)			
Time 1	0.24**	0.20*	0.43***
Time 2	0.21*	0.28**	0.27***
Time 3	0.11	0.30**	0.36***

Note. dYFAS-C 2.0 = Dimensional Yale Food Addiction Scale for Children 2.0. DEBQ-R = Dutch Eating Behavior Questionnaire Restraint Subscale. Asterisks denote significance: * $p < .05$, ** $p < .01$, and *** $p < .001$. Gender (coded 1=male, 2=female).

Supplemental Table S2. Standardized Regression Coefficients from Adjusted Structural Equation Models with Covariates

Path	Predictor	Outcome	*b*	SE	*Z*	*p*	95% CI lower	95% CI upper
1	Food addiction	Dietary restraint	0.23	0.06	3.80	< .001	0.11	0.35
2	Dietary restraint	Food addiction	0.05	0.05	0.90	0.37	-0.06	0.15
3	Food addiction	Food addiction	0.70	0.05	13.59	< .001	0.60	0.80
4	Dietary restraint	Dietary restraint	0.54	0.06	9.12	< .001	0.42	0.66
5	Age	Dietary restraint	0.02	0.05	0.30	.76	-0.09	0.12
6	Gender	Dietary restraint	0.05	0.11	0.42	.67	-0.17	0.23
7	BMI	Dietary restraint	0.10	0.06	1.56	.12	-0.03	0.17
8	Age	Food addiction	0.07	0.05	1.51	.13	-0.02	0.17
9	Gender	Food addiction	0.18	0.09	1.90	.06	-0.01	0.36
10	BMI	Food addiction	-0.02	0.06	-0.31	.76	-0.13	0.09

Model Fit: $X^2(20) = 70.5$ ($p < .01$); SRMR = 0.14; CFI = 0.86. CI = confidence interval
